# Transportation and production collaborative scheduling optimization with multi-layer coding genetic algorithm for non-pipelined wells

**DOI:** 10.1016/j.heliyon.2024.e41307

**Published:** 2024-12-17

**Authors:** Qiushi Li, Yuze Li, Haitong Sun, Wei Song, Honghong Li, Xiaoyong Gao, Chaodong Tan, Yaoyun Liu, Hongbing Liu

**Affiliations:** a Digital and Intelligent Business Unit, PetroChina Changqing Oil Field Branch, Xi^'^an, 710018, China; b Sinopec Gas Storage Branch, Zhengzhou, 450000, China; c Department of Automation, China University of Petroleum, Beijing, 102249, China; d Qinghai Oilfield Company, PetroChina, Qinghai, 817500, China; e Kunlun Digital Technology Co., Ltd, Beijing, 102249, China; f Tianjin Petroleum Vocational and Technical College, Tianjin, 301607, China

**Keywords:** MILP, Genetic algorithm, Quick solution, Minimize the driving distance

## Abstract

The marginal wells in low-permeability oil fields are characterized by small storage size, scattered distribution, intermittent production, etc. The construction of large-scale gathering pipelines has large investment. So the current production mode is featured by single well tank oil storage, oil tank truck transportation and manual tank truck scheduling. At present, oil well production and crude oil transportation scheduling mainly rely on manual formulation, which has poor coordination and seriously restricts the release of oil well production capacity and the reduction of transportation cost. A mixed-integer linear programming (MILP) model representation was proposed in our previous work. The scale of the built model variables is huge, and the model solution time is long. In this paper, a genetic algorithm based on multi-layer coding is proposed. The first layer of the design code is the driving path of the oil tanker, and the second layer is the crude oil loading and unloading amount and the cumulative time. The algorithm expands the search domain by flipping, exchanging and shifting the code. In the case analysis part, the exact algorithm and the genetic algorithm designed in this paper are used to solve cases of different scales (5, 10, 30 and 200 oil wells) respectively, and the correctness and effectiveness of the algorithm are verified. The results show that, compared with the exact algorithm, the genetic algorithm designed in this paper can quickly solve a feasible scheduling scheme under different oil well scales, especially in the case of large-scale (200 wells) oil well groups. The optimal one-time result in the calculation example (200 wells) takes 1062.3 s to run, and the total driving distance of all tank trucks in the obtained feasible scheme is 11280 km. This study can guide oilfields to quickly formulate dispatching plans and minimize travel distances in non-pipeline well tanker dispatching.

## 1 Introduction

**Fig. 1:**
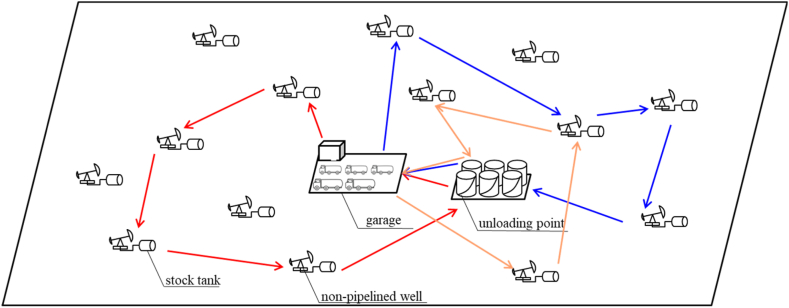
Schematic diagram of Optimization of production and transportation scheduling for non-pipelined wells.

**Fig. 2:**
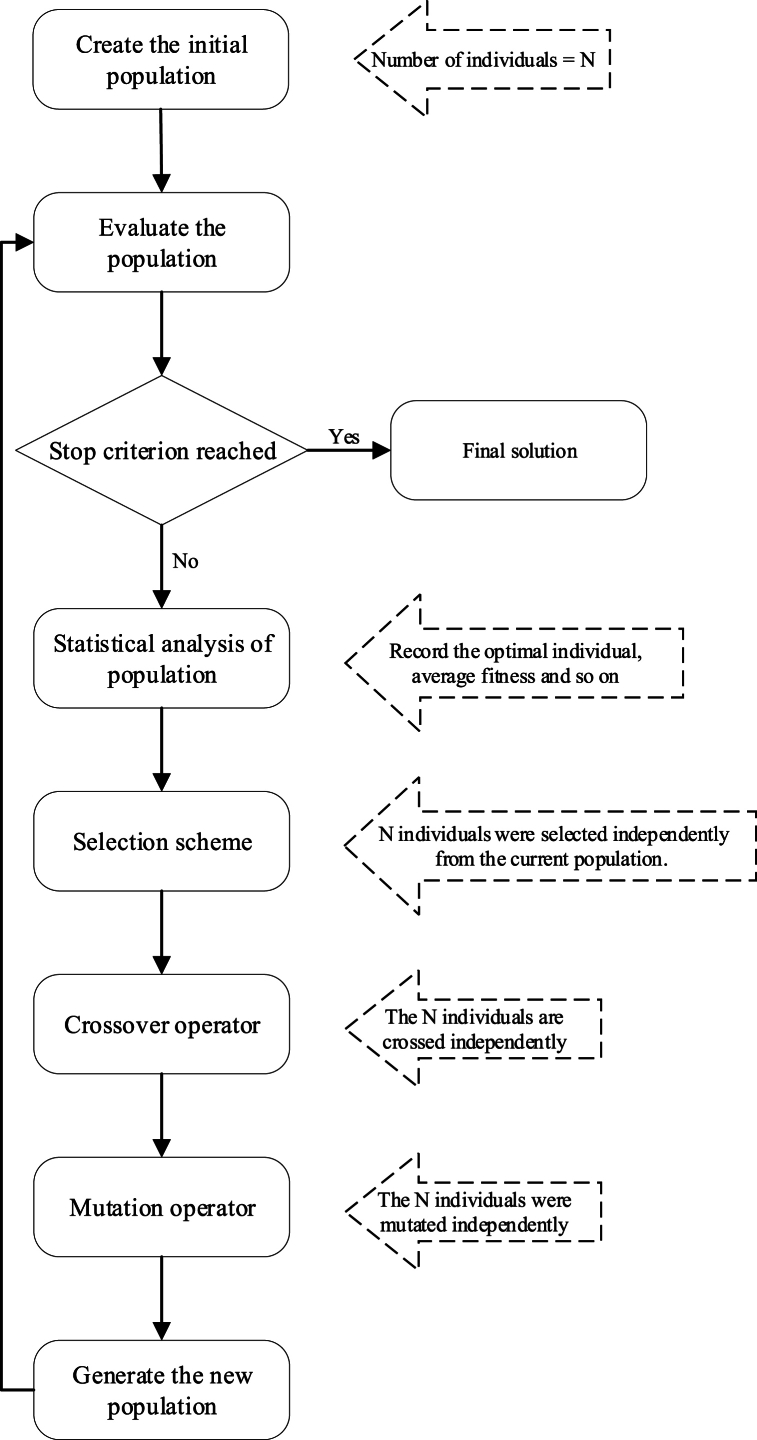
Solution process.

Marginal oil fields characterized by low permeability possess abundant crude oil reserves, and there are a large number of pumping wells. Some of the oil wells are too low in production, sparsely distributed or restricted by geographical conditions. The investment in the construction of oil pipelines is too large, the pipelines are easy to block, and the cost performance is extremely low. Large-scale single-pull wells are widely distributed. For such oil wells, as shown in Fig. 1, a small oil storage tank is set up next to the well, and a fixed or quantitative tank truck is used for storage and transportation, referred to as non-pipelined wells. Crude oil pulling. The development mode using non-pipelined wells is common for marginal oilfield(Zhang et al., 2019; Tan, CD,2022).At present, the scheduling problem is mainly completed by the experience of field experts. When the number of oil wells increases, the number of tank trucks increases, and it becomes extremely difficult to formulate a reasonable dispatch plan based on expert experience, and unscientific scheduling plan will lead to a series of safety accidents in the oil well, such as emergency shutdown of the wellbore due to the overflow of the storage tank, etc. As a result, the production timeliness of oil wells is low and the production potential cannot be fully released, seriously affect oilfield production, production safety and economic benefits. The non-pipelined well group production pull and transportation scheduling model solves the problem of reasonable scheduling of output crude oil (see Fig. 2).

The non-pipelined well group production pull and transportation scheduling model can be described as a Capacitated Truck Routing Problem (CVRP), an extension of VRP. The truck routing problem (VRP) is a classic optimization problem in road transportation and has been widely studied and practiced since it was first introduced by Dantzig and Ramser(1959) [1].In 1964, Clarke and Wright (1964) [2]summarized this problem as a mathematical model with a definite framework, that is, how to use a group of trucks with different capacities to serve a group of customers scattered near a central warehouse. This is the early research of the "Truck Routing Problem" (VRP). Early research on VRP was relatively simple, and the constraints of the model were not complicated. However, with the development of the times and more and more integration with real life, the VRP model has gradually become complicated, and many variants have also been derived [3].For example: Capacitated Truck Routing Problem (CVRP) [4],and CVRP can be divided into non-full-load truck scheduling problem and full-load truck scheduling problem according to the loading task situation. Another example: Truck routing problem with time window (VRPW) with the constraint of the time window of the customer being visited [[5], [6], [7]].In VRPTW, in addition to meeting the limitations of the VRP, the truck must also meet the time window limit of the demand point [8]. The time window limit of the demand point can be divided into two types, one is the hard time window, the other is soft time window [9].These deformations of VRP add considerable complexity to the model. VRP is a NP-hard problem [10].Therefore, the exact algorithm is only valid for small problem instances [11] and heuristic algorithms can generally find relatively good solutions for large-scale problem instances. Because the traditional VRP is a NP-hard problem, the mathematical model involved in this article is clearly a NP-hard problem. Therefore, a heuristic algorithm is utilized to solve the model. In the initial phase of the work, a Mixed-Integer Linear Programming (MILP) model was formulated using a discrete-time representation, which was presented for scheduling the operation of non-pipelined wells. The discrete-time model divides the entire scheduling cycle into equal length and fixed time periods. Each scheduling event occurs on the boundary of a certain time period. Adjacent time periods are connected by various constraint equations. Although the model description can be made more accurate by reducing the time period, blindly shrinking the time period will cause the number of variables in the model to increase exponentially, and the scale of the model variables becomes extremely large. When the actual scale increases, such as the increase in the number of non-pipelined wells and the increase in the number of tank trucks, the variables in the model will also increase. The increase in the number of variables will cause great difficulties for the subsequent model solving, and even lead to no solution to the model. Therefore, attention is turned to the heuristic algorithm.

Through extensive research literature, it is found that some scholars have begun to use heuristic algorithms to solve the problems of the oil industry, and have achieved good results, Yang Liu established a high-dimensional mixed integer nonlinear layout optimization mathematical model involving the pipeline network structure parameters and pipeline design parameters, a combined optimization strategy based on MPSO algorithm, meshing set partitioning method and adjacent position fuzzy set solving method is proposed and analyzed [12]. Qi-Hong Feng has established a well production optimization method using a streamline features-based objective function and the Bayesian Adaptive Direct Search (BADS) algorithm to solve well production optimization models. It has been verified that the BADS algorithm is superior to other commonly used algorithms in terms of convergence speed, solution stability, and optimization accuracy. Furthermore, this method can significantly speed up the optimization process of oil well production [13]. Rui-Zhong Jiang has used a genetic algorithm for well location optimization to determine the optimal number of wells and their respective positions. The genetic algorithm toolbox built into Matlab was utilized to optimize the well locations in specific blocks of the Sulige gas field. This study provides scientific methods and means for optimizing well locations in the Sulige gas field, which helps to improve the recovery rate and economic benefits of the gas field [14]. Arash Javadi has discussed how to combine artificial neural networks and genetic algorithms to optimize gas injection in the oil and gas industry, particularly in case studies for improving oil recovery (EOR) applications. The literature mentions a comparison between genetic algorithm (GA) and particle swarm optimization (PSO) algorithms in optimizing the operating conditions and positions of injection and production wells, as well as the positive impact of these two algorithms on project profitability [15]. Francisco Waldemar Mosqueda Jim é nez has explored how to apply genetic algorithms to optimize integrated petroleum production systems and proposed an optimization model based on oil extraction systems. The results indicate that, compared to traditional node analysis methods, the profit from selling hydrocarbons has increased economically [16]. In terms of vehicle routing, heuristic algorithm has been very mature, which plays a very important leading role in our research and solution of the coupling model of oil production and vehicle routing involved in this paper. Charles J. Malmborg aiming at the truck scheduling problems based on a unit periods of waiting criterion using genetic algorithms to effectively find high-quality scheduling solutions through a limited search space [17].Yang-Byung Park combined the greedy exchange local optimization algorithm and proposed a hybrid genetic algorithm (HGAV) to solve the vehicle scheduling problem with service expiration time and time limit [18].Barrie M. Baker applies genetic algorithm (GA) to the basic vehicle routing problem (VRP), where customers are supplied from a single warehouse and vehicles are limited by weight and travel distance. Each customer is only allowed to use one car. The results obtained by using the hybrid of this GA and neighborhood search method show that this method has more advantages than tabu search and simulated annealing methods in terms of solution time and quality [19]. Franklin T. Hanshar proposed a genetic algorithm (GA) to solve the dynamic vehicle routing problem (DVRP). Compared with the tabu search method and the ACS method, the proposed GA method performs better in minimizing travel costs [20].Bin Yang designed a hybrid genetic algorithm (GA) to solve a multi-objective vehicle routing problem (VRP) model with the goal of reducing costs, improving customer satisfaction and mitigating ecological harm [21].Ali Mohtashami proposed a new method based on dynamic genetic algorithm to minimize the total operation time of dispatching vehicles in the cross-docking system [22].Yiyong Xiao proposed a hybrid solution method combining genetic algorithm and precise dynamic programming program (GA-DP) to solve the time-dependent vehicle routing and scheduling problem with CO2 emission optimization (TD-VRSP-CO2) to determine optimal vehicle scheduling for vehicle routing [23].Jorge García-Álvarez studied the problem of arranging the charging of electric vehicles in real-world charging stations, and proposed a genetic algorithm with customized operators, especially a new chromosome evaluation method based on generating schedules from priority vectors, and verified the convergence of the algorithm and the quality of the obtained solution are improved [24]. Chunlu Wang combined with the non-dominated sorting genetic algorithm to propose a bus scheduling method to deal with traffic congestion [25].Kanchana Sethanan uses a new hybrid differential evolution algorithm with fuzzy logic controller genetic operators to solve the multi-trip vehicle routing problem with backhaul and heterogeneous fleets [26]. Ali Mohtashami proposed a new genetic algorithm based on non-dominated sorting, which is used to simultaneously dispatch vehicles of different types and different capacities in distribution centers working at cross terminals [27].

Based on the studies in the above references, it has been found that genetic algorithms are a promising approach for solving scheduling optimization problems. However, there is a scarcity of relevant research on the application of genetic algorithms in the field of non-pipelined wells transportation scheduling optimization. This paper is motivated to develop efficient solution approaches for the optimization of production and transportation scheduling for non-pipelined wells, with objective of minimizing the total time. The designated model is a derivative problem of the Capacitated Truck Routing Problem (CVRP). The proposed solution algorithm holds substantial significance in reducing transportation costs within oilfield operations. By extending the work of Zhang et al. (2019), the primary contributions encompass a multi-level coding genetic algorithm grounded in a mixed integer linear programming (MILP) model. The multi-level coding genetic algorithm effectively integrates the quantity and location aspects of non-pipeline oil well transportation tasks, applying the genetic algorithm to the optimization scheduling of non-pipeline oil wells. This algorithm overcomes the large number of variables, long solution time or even no solution caused by discrete-time modeling. Based on the original model, we used domestic XX oilfield data for simulation calculations, and compared the solution efficiency and solution results of the precise algorithm and the genetic algorithm. Experimental results show that when the scheduling period becomes longer, the accurate algorithm cannot give a feasible solution within an acceptable time range, and the genetic algorithm designed in this paper can still give a feasible solution in a short time.

The rest of the paper is organized as follows: Section 2 provides a review of pertinent literature; Section 3 revisits the MILP model; Section 4 introduces the proposed genetic algorithm incorporating multi-layer coding; Section 5 presents computational experiments assessing the performance of the proposed algorithm in comparison to prior research; and Section 6concludes the paper.

## 2 Mathematical formulation

First of all, review the model. The purpose of this is to make it easier for us to better explain the constraints of the model when designing the heuristic algorithm for the model, and to make the entire solution process more clear and complete.

### 2.1 Problem description

The objective function of this model is to minimize the total running time of all tank trucks. The model contains two sets of constraints. The first set describes the constraints for the dispatch of tank trucks between different locations (garages, oil wells, and unloading points). The second set of descriptions involves constraints on material balance and storage and handling capacity.

### 2.2 Notation

**Figure:**
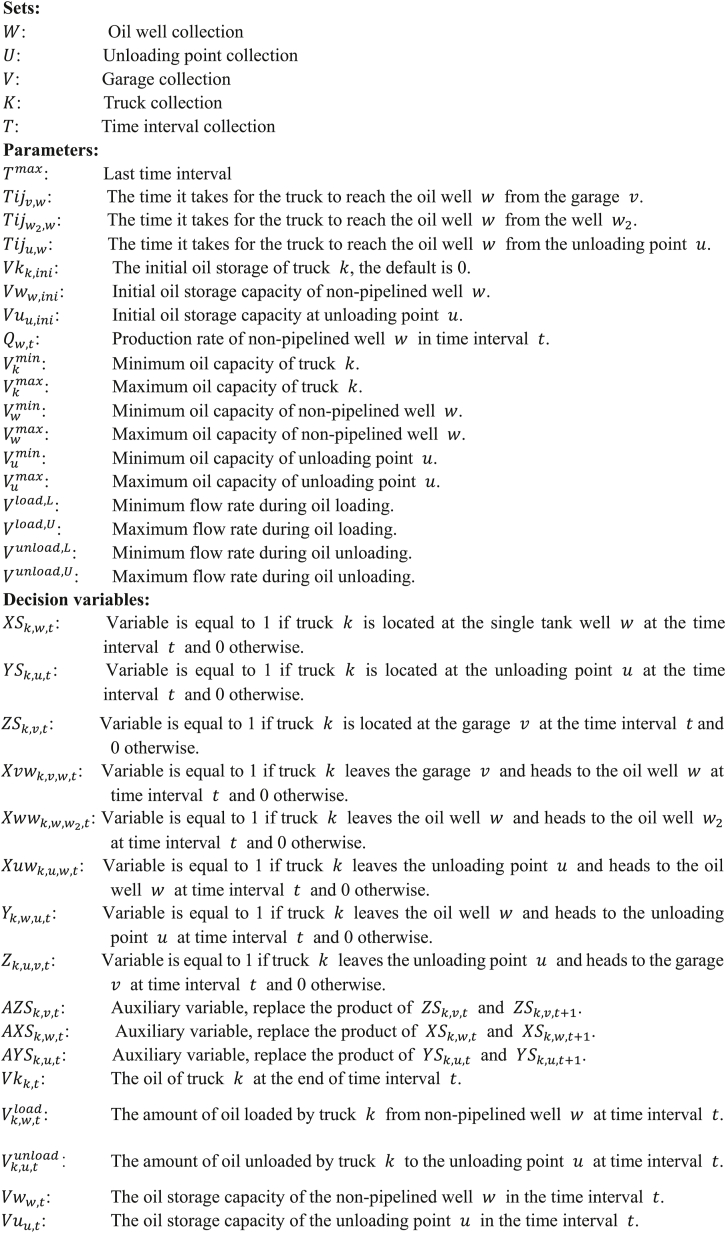

### 2.3 Assumptions

For this problem, the scheduling rules to be followed are listed as follows:

(a) each truck can at most stay in one location at any time
(b) each truck can have at most one task at any time
(c) each well can only be visited by at most one tank truck at any time
(d) the tank truck must be in the garage at the initial and last moment
(e) all loaded oil must be unloaded at the unloading point before the tank truck returns to the garage
(f) mass balance and capacity limit for all tanks (i.e. stock tank for wells, tanks in tank trucks, tanks in oil gathering station) should meet

The following information will be given:

(a) capacity limitations and initial oil reserves of tank trucks, stock tanks and unloading points
(b) the production rate of wells
(c) loading and unloading capacity of tank trucks
(d) flow rate of transporting outwards of unloading points
(e) time required for the tank truck to travel among wells, garages and unloading points
(f) time horizon under considered

The following assumptions are proposed in this paper.

(a) the production rate of oil wells during time horizon is constant
(b) the unloading points transport oil outwards during the whole time horizon at constant speed.

The problem is to determine the following variables:

(a) sequencing and departure time of the tank truck departing to the garages, wells and unloading points
(b) the duration of tank truck's stay in wells, unloading points and garages
(c) amount of oil that tank trucks load at stock tanks or unload at unloading points during each time interval
(d) oil storage quantity of tank trucks, stock tanks and unloading points at each time

### 2.4 Scheduling constraints

In order to describe the arrival and departure actions of tank trucks at different positions and obtain a scheduling plan that meets the actual production needs, some specific constraints need to be introduced.

**Single location constraint.** Any truck can stay in at most one position at any time.

(1) $$\sum_{w}{XS}_{k,w,t}+\sum_{u}{YS}_{k,u,t}+\sum_{v}{ZS}_{k,v,t}\leq 1,\forall k\in K,t\in T$$

(2) $$\sum_{v\in V}ZS_{k,v,1}=1,\forall k\in K$$

(3) $$\sum_{v\in V}ZS_{k,v,T^{\max}}=1,\forall k\in K$$

**Single task constraint.** Any truck can be assigned at most one task at any time.

(4) $$\sum_{v\in V}\sum_{w\in W}Xvw_{k,v,w,t}+\sum_{w_{2}\in W}\sum_{w\in W}Xww_{k,w_{2},w,t}+\sum_{u\in U}\sum_{w\in W}Xuw_{k,u,w,t}+\sum_{w\in W}\sum_{u\in U}Y_{k,w,u,t}+\sum_{u\in U}\sum_{v\in V}Z_{k,u,v,t}\leq 1,\forall k\in K,t\in T$$

(5) $$Xww_{k,w2,w,t}=0,\forall k\in K,w\in W,t\in T$$

**Single access constraint.** Any oil well can be visited by at most one truck at any time.

(6) $$\sum_{k}XS_{k,w,t}\leq 1,\forall w\in W,t\in T$$

**Dispatch variable constraints.** The above three types of constraints describe the basic scheduling rules that need to be followed.

**The truck leaves the restraint.** Consider the process of an oil truck leaving the garage.

(7) $$ZS_{k,v,t}\left(ZS_{k,v,t}-ZS_{k,v,t+1}\right)=\sum_{w\in W}Xvw_{k,v,w,t}\;\forall k\in K,v\in V,t\leq T^{\max}-1$$

(8) $$XS_{k,w,t}\left(XS_{k,w,t}-XS_{k,w,t+1}\right)=\sum_{w2\in W}Xww_{k,w,w2,t}+\sum_{u\in U}Y_{k,w,u,t}\;\forall k\in K,w\in W,t\leq T^{\max}-1$$

(9) $$YS_{k,u,t}\left(YS_{k,u,t}-YS_{k,u,t+1}\right)=\sum_{w\in W}Xuw_{k,u,w,t}\;\forall k\in K,u\in U,t\leq T^{\max}-1$$

**The truck reaches the restraint.** Consider the process of a truck truck arriving at a non-pipelined well.

(10) $$\sum_{v\in V}Xvw_{k,v,w,{t-Tij}_{v,w}}+\sum_{w_{2}\in W}Xww_{k,w_{2},w,t-{Tij}_{w_{2},w}}+\sum_{u\in U}Xuw_{k,u,w,{t-Tij}_{u,w}}=XS_{k,w,t}\left(XS_{k,w,t}-XS_{k,w,t-1}\right)\;\forall k\in K,u\in U,t\in T,t\geq {Tij}_{v,w},t\geq {Tij}_{w_{2},w},t\geq {Tij}_{u,w}$$

(11) $$\sum_{w\in W}Y_{k,w,u,t-{Tij}_{w,u}}=YS_{k,u,t}\left(YS_{k,u,t}-YS_{k,u,t-1}\right)\;\forall k\in K,u\in U,t\in T,t\geq {Tij}_{w,u}$$

(12) $$\sum_{w\in W}Z_{k,u,v,t-{Tij}_{u,v}}=ZS_{k,v,t}\left(ZS_{k,v,t}-ZS_{k,v,t-1}\right)\;\forall k\in K,v\in V,t\in T,t\geq {Tij}_{u,v}$$

### 2.5 Linearization

**Bilinear term.** Constraints (7), (8), (9), (10), (11), (12) involve a number of bilinear terms. Now the constraints containing bilinear terms are transformed into linear constraints according to a specific method.

(13) $${AZS}_{k,v,t}\leq {ZS}_{k,v,t}\;\forall k\in K,v\in V,t\in T$$

(14) $${AZS}_{k,v,t}\leq {ZS}_{k,v,t+1}\;\forall k\in K,v\in V,t\in T,t\leq T^{\max}-1$$

(15) $${AZS}_{k,v,t}\geq {ZS}_{k,v,t}+{ZS}_{k,v,t+1}-1\;\forall k\in K,v\in V,t\in T,t\leq T^{\max}-1$$

(16) $${AZS}_{k,v,t}\geq 0\;\forall k\in K,v\in V,t\in T$$

(17) $${ZS}_{k,v,t}-{AZS}_{k,v,t}=\sum_{v\in V}Xvw_{k,v,w,{t-Tij}_{v,w}}\;\forall k\in K,v\in V,t\in T$$

(18) $${XS}_{k,w,t}-{AXS}_{k,w,t}=\sum_{w_{2}\in W}{Xww}_{k,w,w_{2},t}+\sum_{u\in U}Y_{k,w,u,t}\;\forall k\in K,w\in W,t\in T$$

(19) $${YS}_{k,u,t}-{AYS}_{k,u,t}=\sum_{w\in W}{Xuw}_{k,u,v,t}+\sum_{v\in V}Z_{k,u,v,t}\;\forall k\in K,u\in U,t\in T$$

(20) $$\sum_{u\in U}Z_{k,u,v,t-T_{u,v}}={ZS}_{k,v,t}-{AZS}_{k,v,t-1}\;\forall k\in K,v\in V,t\geq T_{u,v}$$

(21) $$\sum_{w\in W}Y_{k,w,u,t-T_{w,u}}={YS}_{k,u,t}-{AYS}_{k,u,t-1}\;\forall k\in K,u\in U,t\in T,t\geq T_{w,u}$$

(22) $$\sum_{v\in V}{Xvw}_{k,v,w,t-{Tij}_{v,w}}+\sum_{w_{2}\in W}{Xww}_{k,w_{2},t-{Tij}_{w_{2},w}}+\sum_{u\in U}{Xuw}_{k,u,w,t-{Tij}_{u,w}}={XS}_{k,w,t}-{AXS}_{k,w,t-1}\;\forall k\in K,w\in W,t\in T,t\geq {Tij}_{v,w},t\geq {Tij}_{w_{2},w},t\geq {Tij}_{u,w}$$

### 2.6 Production constrains

**Material balance of tank trucks and non-pipelined well.**

When t=1:

(23) $${Vk}_{k,1}={Vk}_{k,ini}+\sum_{w\in W}V_{k,w,1}^{load}-\sum_{u\in U}V_{k,u,1}^{unload}\;\forall k\in K$$

When t≥2:

(24) $${Vk}_{k,t}={Vk}_{k,t-1}+\sum_{w\in W}V_{k,w,t}^{load}-\sum_{u\in U}V_{k,u,t}^{unload}\;\forall k\in K,t\in T,t\geq 2$$

**Material balance for non-pipelined well cans.**

When t=1:

(25) $${Vw}_{w,1}={Vw}_{w,ini}+Q_{w,1}-\sum_{k\in K}V_{k,w,1}^{load}\;\forall w\in W$$

When t≥2:

(26) $${Vw}_{w,t}={Vw}_{w,t-1}+Q_{w,t}-\sum_{k\in K}V_{k,w,t}^{load}\;\forall w\in W,t\in T,t\geq 2$$

**The material balance of the oil tank at the unloading point.**

When t=1:

(27) $${Vu}_{u,1}={Vu}_{u,ini}+\sum_{k\in K}V_{k,u,1}^{unload}\;\forall u\in U$$

When t≥2:

(28) $${Vu}_{u,t}={Vu}_{u,t-1}+\sum_{k\in K}V_{k,u,t}^{unload}\;\forall u\in U,t\in T,t\geq 2$$

**The total amount of loading and unloading oil is balanced.**

(29) $$\sum_{w\in W}\sum_{t\in T}V_{k,w,t}^{load}=\sum_{u\in U}\sum_{t\in T}V_{k,u,t}^{unload}\;\forall k\in K$$

**Storage capacity constraints.** The oil load or storage capacity under consideration must be between the specified maximum and minimum values.

(30) $$V_{k}^{\min}\leq V_{k,t}\leq V_{k}^{\max}\;\forall k\in K,t\in T$$

(31) $$V_{w}^{\min}\leq V_{w,t}\leq V_{w}^{\max}\;\forall w\in W,t\in T$$

(32) $$V_{u}^{\min}\leq V_{u,t}\leq V_{u}^{\max}\;\forall u\in U,t\in T$$

**Loading and unloading flow rate limitation.** Restrict the maximum and minimum flow velocity during oil loading and unloading.

(33) $$\left({XS}_{k,w,t}-\sum_{u\in U}Y_{k,w,u,t}-\sum_{w_{2}\in W}{Xww}_{k,w,w_{2},t}\right)V^{load,L}\leq V_{k,w,t}^{load}\leq \left({XS}_{k,w,t}-\sum_{u\in U}Y_{k,w,u,t}-\sum_{w_{2}\in W}{Xww}_{k,w,w_{2},t}\right)V^{load,U}\;\forall k\in K,w\in W,t\in T$$

(34) $$\left({YS}_{k,u,t}-\sum_{w\in W}{Xuw}_{k,u,w,t}-\sum_{v\in V}Z_{k,u,v,t}\right)V^{unload,L}\leq V_{k,u,t}^{unload}\leq \left({YS}_{k,u,t}-\sum_{w\in W}{Xuw}_{k,u,w,t}-\sum_{v\in V}Z_{k,u,v,t}\right)V^{unload,U}\;\forall k\in K,u\in U,t\in T$$

### 2.7 Objective function

The objective function is to minimize the total running time of all tank trucks.

(35) $$minf=\min\sum_{k\in K}\sum_{t\in T}\left(\sum_{v\in V}\sum_{w\in W}{Tij}_{v,w}{Xvw}_{k,v,w,t}+\sum_{w\in W}\sum_{w_{2}\in W}{Tij}_{w,w_{2}}{Xww}_{k,w,w_{2},t}+\sum_{u\in U}\sum_{w\in W}{Tij}_{u,w}{Xuw}_{k,u,w,t}+\sum_{w\in W}\sum_{u\in U}{Tij}_{w,u}Y_{k,w,u,t}+\sum_{u\in U}\sum_{v\in V}{Tij}_{u,v}Z_{k,u,v,t}\right)$$

## 3 Genetic algorithm

The genetic algorithm is a well-known meta-heuristic algorithm, following the natural evolution processes [28].It is a computational model simulating the evolutionary process of Darwin's genetic selection and natural selection, which was first proposed by Professor John H. Holland of Michigan University in 1975 [29]. It uses simple coding technology to represent various complex structures, and guides learning and determining search through simple genetic operation and natural selection of survival of the fittest. Because he uses the way of population to organize the search, he can search multiple regions in the solution space at the same time. Moreover, the search method of population organization makes genetic algorithm especially suitable for large-scale parallel. While endowing genetic algorithm with the characteristics of self-organization, self-adaptive and self-learning, the natural selection of survival of the fittest and simple genetic operation make genetic algorithm free from the constraints of its search space constraints (such as differentiability, continuity, unimodal, etc.), no other auxiliary information (such as derivative), and the search process is not easy to fall into local optimum. These new characteristics make the genetic algorithm not only obtain higher efficiency, but also have the characteristics of simple, easy to operate and universal. Genetic algorithms are compatible to a wide application of different complex optimization problems [30,31].

### 3.1 Solution encoding

Before applying GA, design suitable chromosomes that represent candidate solutions, because this step is the key to the successful implementation of GA.

**Fig. 3:**
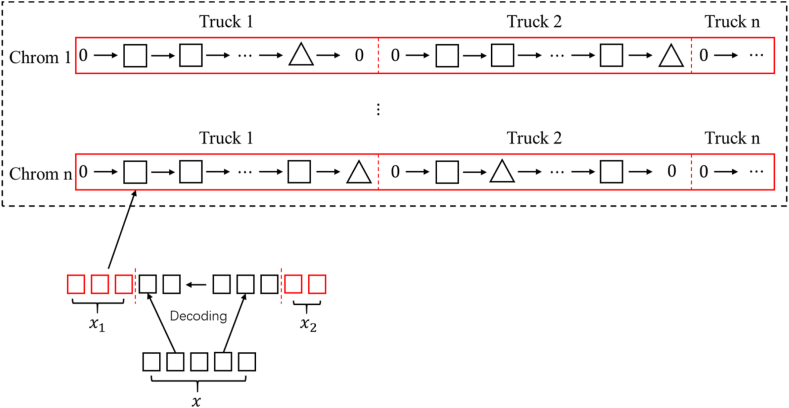
Coding method of vehicle path.

For the MILP model, designed a new chromosome encoding method. Define the task variable of a truck as $B_{n}$ ,that is, a certain truck from one non-pipelined well to the next non-pipelined well or waiting in place at a non-pipelined well is regarded as a task. Each chromosome is composed of several decision variables after decoding, each decision variable in the chromosome represents the current position of the tank truck, the first $B_{n}$ variables represent the first one, and so on. These decision variables constitute represent candidate solutions mapping. The coding method of chromosome is shown in Fig. 3:

Among them, each gene on the chromosome represents a position, which is obtained by decoding the decision variable x, 0 represents the garage, ◻ represents the oil well, △ represents the oil unloading point. The specific decoding process will be described in detail in the **Decision variables** section below.

#### 3.1.1 Encoding rules

In this section, combine the objective function and constraints of the model to explain the above coding rules.

For scheduling constraints, constraints (1), (4) are satisfied naturally. We stipulate that all trucks start from the garage and return to the garage after completing all tasks, so as to meet constraints (2), (3). For constraint (5), it can be described as follows: if several consecutive tasks of a truck are all oil loading operations in the same well, then carry out the elimination of continuous single can pulling operation, that is, merge the same well number in the chromosome and stack the oil loading amount. For constraint (6), it can be described as: when a car needs to go to a single pulling well to load oil, there is a car in the well to load oil, then the later car needs to wait for the front car to finish loading oil before loading oil. In this way, the waiting time will be generated, which will be reflected in the following description of the decision variables in the chromosome. For constraints (7), (8), (9), (10), (11), (12), it has been stipulated that all trucks start from the garage and return to the garage at last. The decision variable in chromosome only indicates that the location of the truck is a well or waiting, so constraints (7), (8), (9), (10), (11), (12) are satisfied. In addition to the scheduling constraints listed in the model, the timeout penalty constraint is also involved in the algorithm design, that is, if the total number of scheduling time periods exceeds the specified number of time periods, the penalty time should be added to the final objective function value.

For production constraints, constraints (23), (24), (25), (26), (27), (28), (29) are satisfied naturally. For constraint (30), we stipulate that if the remaining tank capacity of an oil tanker can not meet the requirement, the oil tanker will go to the unloading point to unload oil first when it goes to the next single pull tank. This point will be explained in detail in the next section of decision variables. The constraints (31), (32) are used as penalty constraints when designing the algorithm, that is, if there is a single tank pulling or overflow at the unloading point in the scheduling process, the penalty time should be added to the final objective function value. In order to ensure the flexibility of the model, the loading and unloading oil flow rate constraint is set to the range value in the original model, but in practical application, the loading and unloading oil flow rate will take different values (fixed value) according to the field application. In this paper, in order to maximize the operation efficiency of the model, take the maximum value of loading and unloading oil flow rate.

#### 3.1.2 Decision variables

Define each decision variable in the chromosome as x before decoding, x is composed of 5 digits from 00000 to 99999.The decoding formulas are (36) and (37).

(36) $$x_{1}=\left(\;x|100\;\right)\mathrm{mod}\;\left(Wn+1\right)$$

(37) $$x_{2}=\left(x\;\mathrm{mod}\;100+1\right)/10$$

formula (36) expresses that the decision variable $x_{1}$ is obtained by decoding. x divided by 100 rounded, take out the first 3 bits of x, divide the obtained 3-digit number by (Wn+1) and take the remainder, the result is 0,1,2,...,Wn.Whenever it encounters a multiple of Wn+1,it is 0, indicates that the truck is waiting, corresponding to $x_{2}$ is the waiting time. In other cases it is 1,2,...,Wn, that is, the serial number of the well participating in the dispatch, corresponding to $x_{2}$ is the amount of oil. In particular, we define if each truck exceeds the maximum capacity of the truck when it goes to the next well to pull oil, the truck will first go to the unloading point to unload the oil. Therefore, taking (Wn+1) in equation (35) to get the serial number of the well 1,2,...,Wn,does not include the oil discharge point.

formula (37) expresses that the decoding obtains the decision variable $x_{2}$.Divide x by 100 and take the remainder, take out the last 2 digits of x, 0,1,2,...,99,add 1 to this number and divide by 10, get another number 0.1,0.2,...,10. If $x_{1}$ is equal to 0, then $x_{2}$ represents the waiting time, if $x_{1}$ is not equal to 0, then $x_{2}$ represent the amount of oil. It is noted that the number 1 is added after dividing x by 100, so that the truck will not be filled with oil when the oil level is 0.

**Fig. 4:**
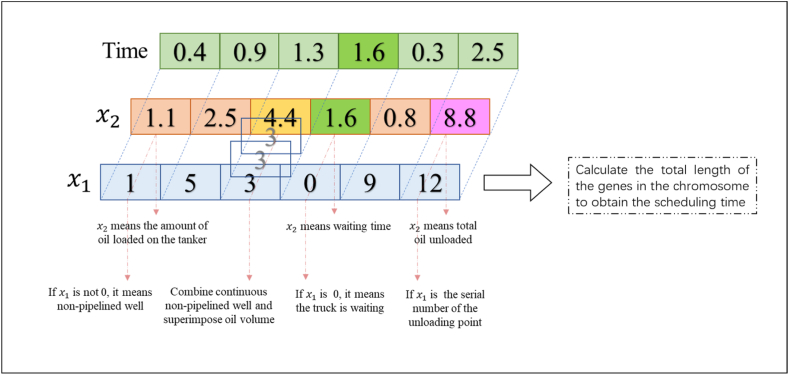
Examples of decoding rules.

The specific decoding example of the chromosome is shown in Fig. 4:

### 3.2 Genetic operators

This study adopts an elite retention strategy, which can ensure that at least one better individual is stably passed on from the previous generation to the next generation in the evolutionary process.

Divide the chromosomes into groups of 4, determine an optimal chromosome for each group, and directly enter the next generation. The other three chromosomes are flipped, exchanged, and shifted based on this optimal chromosome to obtain new children. Generation, the obtained 3 new progeny and the original 1 optimal chromosome enter the next generation.

#### 3.2.1 Flip:

**Fig. 5:**
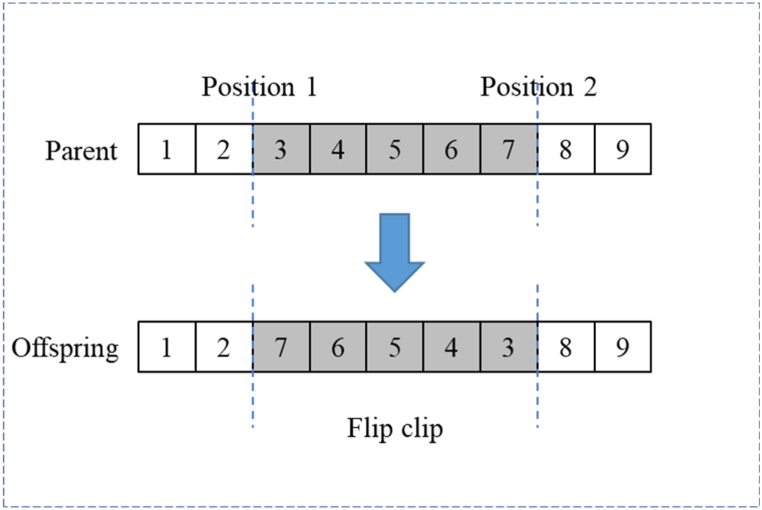
Flip operation.

Two gene positions on the parent chromosome are randomly selected, and the fragments in the middle of the two positions are flipped to form a new daughter chromosome. A specific example is shown in Fig. 5.

#### 3.2.2 Exchange:

**Fig. 6:**
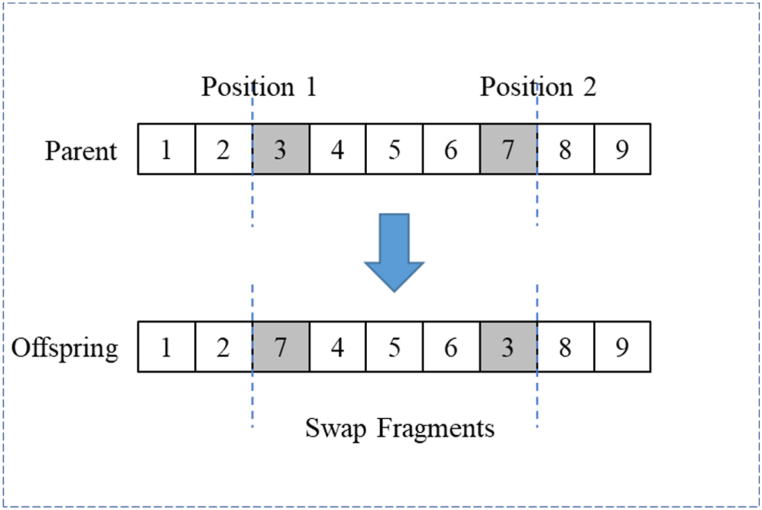
Swap operation.

Two gene positions on the parent chromosome are randomly selected, and the genes corresponding to these two positions are exchanged to form a new daughter chromosome. A specific operation example is shown in Fig. 6.

#### 3.2.3 Displacement:

**Fig. 7:**
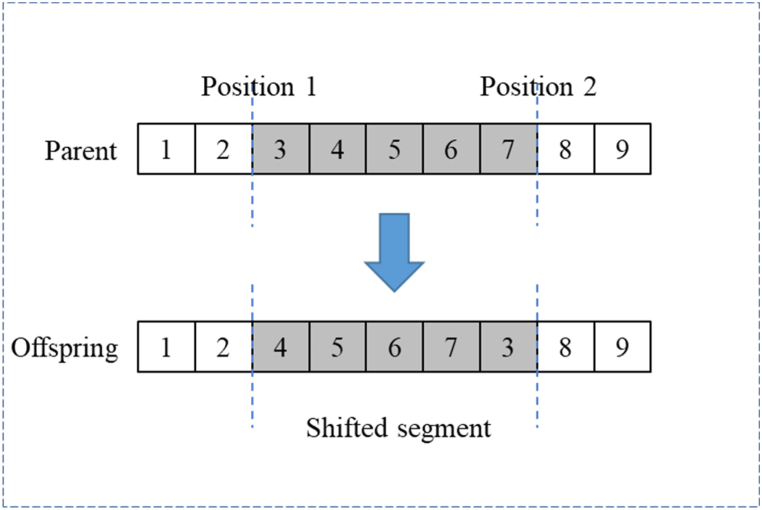
Displacement operation.

Randomly select two gene positions on the parent chromosome, and move the gene corresponding to position 1 to position 2 to form a new daughter chromosome. A specific operation example is shown in Fig. 7.

### 3.3 Fitness function and penalty function

Take the shortest time the vehicle travels as the fitness function, denoted as f(x), which is mapped to the code as the sum of the distances between the decision variables in each chrom.

**Fig. 8:**
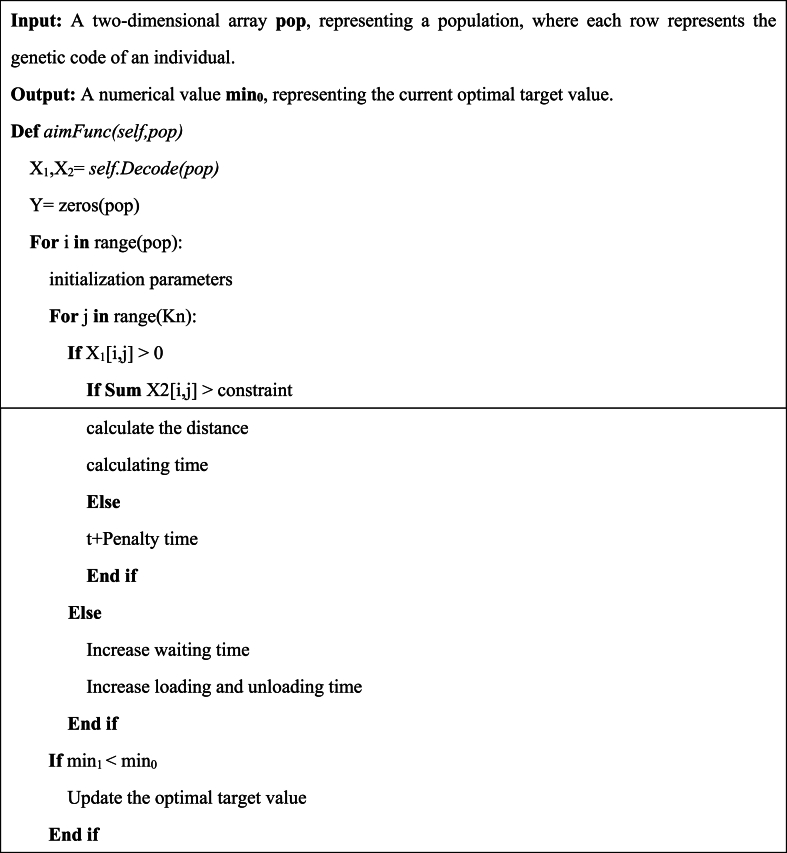
Pseudo-code of the production and pull dispatch.

The penalty function consists of two parts, as shown in formula (38), the first part is in the scheduling process, when the tank capacity of a single pull tank exceeds the maximum tank capacity, a penalty time $t_{1}$ is added to the fitness function; the second part is in the scheduling process, When the unloading point exceeds the maximum tank capacity, a penalty time $t_{2}$ is added to the fitness function. Here $t_{1}$ and $t_{2}$ are set to quite large numbers, which is to make it easier to judge whether the result of the subsequent operation violates the constraint and is punished. The calculation process is shown in Fig. 8.

(38) $$\left\{ \begin{array}{c} f\left(x\right)+t_{1}\;if\;{Vw}_{w,t}>V_{w}^{\max}\\ f\left(x\right)+t_{2}\;if\;{Vu}_{u,t}>V_{u}^{\max} \end{array}\right.$$

## 4 Computational experiments

In this section, the China XX oil field is used as the production scheduling scenario of this model. In this scenario, the solver (precise algorithm) is called and the genetic algorithm designed in this paper is used to solve the model. We set up cases with different gradient scales for simulation calculation. The case scale and parameter settings are shown in Table 1. According to the characteristics of the discrete time model, considering the balance between the solution time and the accuracy of the model, the time range is evenly divided into 672 time intervals(7 days), and each time interval represents 15 min. The maximum running time of the model is set to 28800 s(8h),this time is the maximum solution time acceptable in engineering. The performance of using GA to solve the model is measured according to the accurate method (Gurobi) of the original model based on the MILP model. In the simulation experiment, we used Gurobi and GA to solve the cases of different scales, and compared the solution results. In terms of parameter setting, set some tank trucks to the same specifications, and the maximum fuel capacity of the vehicle is 10 m^3^. The maximum capacity of a single pull tank is 20 m^3^, the minimum storage capacity of the unloading point is 200 m^3^, the maximum storage capacity of the unloading point is 5000 m^3^, the lower limit of vehicle filling speed is 0m^3^/15min, the upper limit of vehicle filling speed is 5m^3^/15min, and the lower limit of vehicle unloading speed is 0 m^3^/15min. The upper limit of the unloading speed is 10m^3^/15min, the initial fuel load of the vehicle is 0 m^3^, and the initial fuel storage at the unloading point is 2000 m^3^. Number all dispatching elements, the garage is number 0. The initial oil storage capacity of a single well tank is shown in Table 1.

**Table 1:** Non-pipelined wells data.

Case number	Number of garages	Number of wells	Number of tanker trucks	Single pull can	Tank truck capacity	Oil discharge point capacity	Oil filling rate	Oil discharge rate (m^3^/d)
1	1	5	2	20	10	5000	5	10
2	1	10	3	20	10	5000	5	10
3	1	30	6	20	10	5000	5	10
4	1	200	20	20	10	5000	5	10

### 4.1 Exact algorithm solution

First use an accurate algorithm to solve the case. This case was programmed by commercial software Python 3.70, and the solution was run on a workstation with Intel Xeon E5-2630 v4 @2.2 GHz, RAM 128.0 GB, and 64-bit Win10 operating system. The MILP model is solved by the Gurobi 9.1.2 solver and stops when the first feasible solution is obtained [32]. The specific solution results are shown in Table 2. The table lists the model analysis results (number of constraints, number of variables, and preprocessing time), solution time, and final solution results for four different cases. From the solution results of the precise algorithm, it can be seen that in small-scale problems (Case 1∼Case 3), when the scheduling cycle is relatively short, a feasible solution, even the optimal solution, can be obtained by using the precise algorithm. With the increase of the scheduling cycle, the solution time of the model gradually increases, until a feasible solution cannot be obtained in an acceptable time. When the scale of the model increases (Case 4), even in a short period, the accurate algorithm cannot find a feasible solution in an acceptable time range When the model cannot find a feasible solution, the oilfield cannot formulate a reasonable scheduling scheme, which is very fatal in engineering applications.

**Table 2:** Case statistics and results.

	Schedule time	Number of constraints	Number of continuous variables	Number of binary variables	Pretreatment time	CPU time	Number of iterations	Objective function
Case 1	1	14406	480	10,560	0.1	8	32,005	165
	3	43,398	1440	31,680	0.24	2343.6	7,851,124	210
	7	101,382	3360	73,920	20.46	18,000	22,070,800	480
Case 2	1	35,280	960	44,640	1.81	630.8	4,739,765	225
	3	106,320	2880	133,920	26.6	4400	3,264,916	303
	7	248,400	6720	312,480	156.5	18,000	–	–
Case 3	1	176,952	2880	607,680	41.3	18,000	8,250,640	345
	3	533,496	8640	1,878,624	719.1	18,000	1,188,057	–
	7	12,346,584	20,161	4,253,760	5275.7	18,000	236,950	–
Case 4	1	–	–	–	–	18,000	–	–
	3	–	–	–	–	18,000	–	–
	7	–	–	–	–	18,000	–	–

### 4.2 Genetic algorithm solution

The initial population size, iteration number and penalty value of the genetic algorithm in Table 3 are set according to the model size. When the model size is small, a smaller initial population size and fewer iterations are sufficient to obtain a feasible solution. The t in the penalty value is the penalty coefficient, and the value of t is generally required to be large enough, but in this study, if the value is too large, the solution efficiency will decrease. Therefore, for the model in this paper, the penalty value only needs to be required to exceed the value obtained by the solution. When the penalty value is greater than the feasible solution obtained from the solution, it is considered that the solution process does not trigger the default penalty, that is, all constraints are satisfied, and a reasonable solution is finally obtained. The specific solution results are shown in Table 3.

**Table 3:** Genetic algorithm parameter settings and solution results.

	Scheduling period	Initial population	Number of iterations	Penalty value	Solving time	Number of vehicles used	Solve results
Case 1	7 days	100	50	+(f(x)+750)	3.8	1	630
Case 2	7 days	400	200	+(f(x)+1500)	72.4	1	1215
Case 3	7 days	400	100	+(f(x)+3000)	81.3	2	1560
Case 3	7 days	400	200	+(f(x)+3000)	90.1	2	1620
Case 3	7 days	600	200	+(f(x)+3000)	160	4	2018
Case 3	7 days	600	200	+(f(x)+3000)	107	3	1470
Case 4	7 days	600	200	+(f(x)+15000)	987.5	6	12,195
Case 4	7 days	600	200	+(f(x)+15000)	1371.3	7	13,125
Case 4	7 days	800	200	+(f(x)+15000)	1213.7	6	11,880
Case 4	7 days	800	200	+(f(x)+15000)	1062.3	5	11,280

**Fig. 9:**
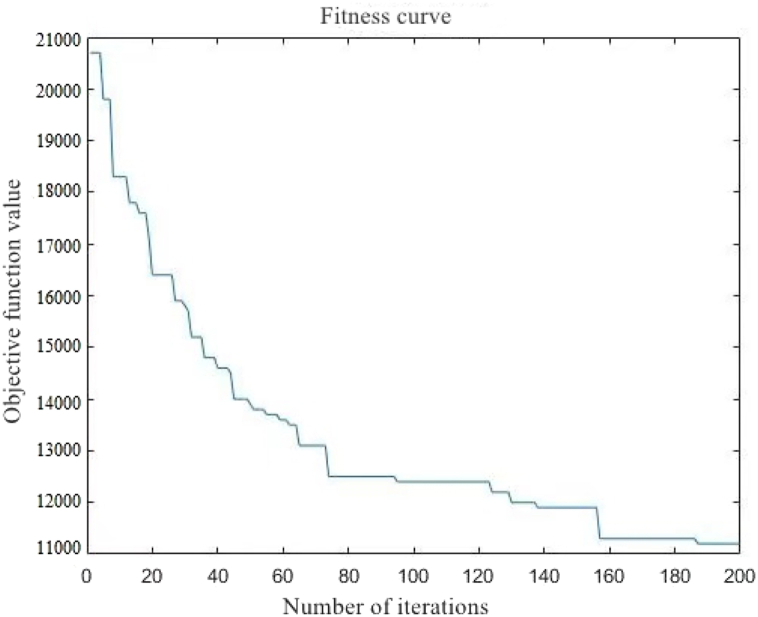
Fitness curve.

Among the solved cases, Case 4 has the largest scale and is the most representative for the large-scale problem studied in this chapter. Select a specific analysis with the best results in the example. The optimal one-time result in the calculation example takes 1062.3 s to run, and the total driving distance of all tank trucks in the obtained feasible scheme is 11280 km. The fitness curve during the calculation process is shown in Fig. 9.

**Fig. 10:**
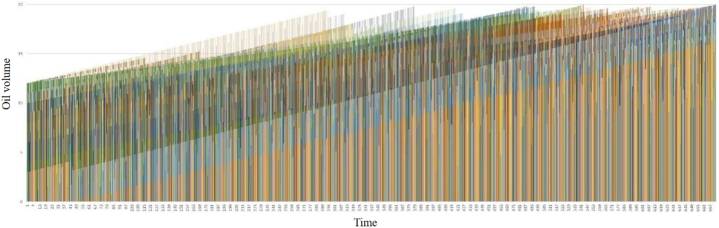
The oil volume of each non-pipelined well changes with time.

Fig. 10 shows the oil volume change of each single pull tank during the whole dispatching process, each color represents an oil well, the abscissa represents time, and the ordinate represents oil volume. In the case setting, we set the maximum capacity of a single pull tank to 20 m^3^. From Fig. 7, it can be observed that during the scheduling process, all single pull tanks are overflowing tanks, which meet the constraints of the model.

It can be seen from Table 3 that the genetic algorithm designed for the optimization model of production and transportation scheduling for non-pipelined and transportation wells can give a feasible solution in a short time. It effectively solves the problem that the exact algorithm takes a long time to solve the small-scale long-period scheduling problem and the large-scale long-period scheduling problem or cannot give a feasible solution within an acceptable time range. There is no tank overflow in each oil well and oil discharge point, and the total scheduling time does not exceed the specified time. Therefore, the genetic algorithm designed in this paper has achieved good results in the optimization of production and transportation scheduling for non-pipelined and transportation wells.

## 5 Conclusions

In this article, the heuristic algorithm for solving the optimization of production and transportation scheduling for non-pipelined wells is studied. For small-scale problems, you can use the MIP solver to directly solve the MILP model to the optimum. For large-scale problems, accurate algorithms cannot give a feasible solution within a reasonable time required by the actual problem. A genetic algorithm with multi-layer coding is proposed for solving large-scale practical problems. The first layer of the design code is the driving path of the tanker, and the second layer is the crude oil loading and unloading amount and the accumulated time. Cases with different scale gradients are set for simulation calculation. Computational experiments show that the proposed method is effective and efficient, and can quickly give a reasonable scheduling plan. The advantage is particularly obvious in the case of large-scale oil well groups. In the case of 200 wells, with reasonable population size and number of iterations, the optimal one-time result in the calculation example takes 1062.3 s to run, and the total driving distance of all tank trucks in the obtained feasible scheme is 11280 km.This study can guide oilfields to quickly formulate dispatching plans and minimize travel distances in non-pipeline wells tanker dispatching.

Obviously, the model and solution research of this scenario is far more than that. Future research on this topic may focus on the following directions: By combining exact algorithms with heuristics. A feasible solution is quickly obtained through a heuristic algorithm, and then the feasible solution is input into the accurate algorithm as an initial solution, so that it can continue to search downwards on this basis, so as to obtain a better solution or even an optimal solution faster. Combining multiple heuristic algorithms, such as particle swarm algorithm or tabu search algorithm, makes the advantages of the algorithms complement each other, so that a better scheduling scheme can be obtained faster.

## CRediT authorship contribution statement

**Qiushi Li:** Writing – original draft, Investigation. **Yuze Li:** Writing – original draft, Methodology. **Haitong Sun:** Writing – review & editing, Data curation. **Wei Song:** Methodology, Data curation. **Honghong Li:** Formal analysis. **Xiaoyong Gao:** Methodology. **Chaodong Tan:** Writing – review & editing, Resources, Methodology. **Yaoyun Liu:** Data curation. **Hongbing Liu:** Data curation.

## Declaration of Competing Interest

The authors declare that they have no known competing financial interests or personal relationships that could have appeared to influence the work reported in this paper.
